# The choroid plexus sodium‐bicarbonate cotransporter NBCe2 regulates mouse cerebrospinal fluid pH

**DOI:** 10.1113/JP275489

**Published:** 2018-08-12

**Authors:** Henriette L. Christensen, Dagne Barbuskaite, Aleksandra Rojek, Hans Malte, Inga B. Christensen, Annette C. Füchtbauer, Ernst‐Martin Füchtbauer, Tobias Wang, Jeppe Praetorius, Helle H. Damkier

**Affiliations:** ^1^ Department of Biomedicine, Health Aarhus University Denmark; ^2^ Department of Cellular and Molecular Medicine, Faculty of Health and Medical Sciences University of Copenhagen Denmark; ^3^ Department of Bioscience, Science and Technology Aarhus University Denmark; ^4^ Department of Molecular Biology and Genetics, Science and Technology Aarhus University Denmark

**Keywords:** NBCe2, choroid plexus, cerebrospinal fluid, respiratory acidosis, pH regulation

## Abstract

**Key points:**

- Normal pH is crucial for proper functioning of the brain, and disorders increasing the level of CO_2_ in the blood lead to a decrease in brain pH.
- CO_2_ can easily cross the barriers of the brain and will activate chemoreceptors leading to an increased exhalation of CO_2_.
- The low pH, however, is harmful and bases such as HCO_3_^−^ are imported across the brain barriers in order to normalize brain pH.
- We show that the HCO_3_^−^ transporter NBCe2 in the choroid plexus of the blood‐cerebrospinal fluid barrier is absolutely necessary for normalizing CSF pH during high levels of CO_2_.
- This discovery represents a significant step in understanding the molecular mechanisms behind regulation of CSF pH during acid‐base disturbances, such as chronic lung disease.

**Abstract:**

The choroid plexus epithelium (CPE) is located in the brain ventricles where it produces the majority of the cerebrospinal fluid (CSF). The hypothesis that normal brain function is sustained by CPE‐mediated CSF pH regulation by extrusion of acid‐base equivalents was tested by determining the contribution of the electrogenic Na^+^‐HCO_3_^−^ cotransporter NBCe2 to CSF pH regulation. A novel strain of NBCe2 (*Slc4a5*) knockout (KO) mice was generated and validated. The base extrusion rate after intracellular alkalization was reduced by 77% in NBCe2 KO mouse CPE cells compared to control mice. NBCe2 KO mice and mice with CPE‐targeted NBCe2 siRNA knockdown displayed a reduction in CSF pH recovery during hypercapnia‐induced acidosis of approximately 85% and 90%, respectively, compared to control mice. NBCe2 KO did not affect baseline respiration rate or tidal volume, and the NBCe2 KO and wild‐type (WT) mice displayed similar ventilatory responses to 5% CO_2_ exposure. NBCe2 KO mice were not protected against pharmacological or heating‐induced seizure development. In conclusion, we establish the concept that the CPE is involved in the regulation of CSF pH by demonstrating that NBCe2 is necessary for proper CSF pH recovery after hypercapnia‐induced acidosis.

## Key points

- Normal pH is crucial for proper functioning of the brain, and disorders increasing the level of CO_2_ in the blood lead to a decrease in brain pH.
- CO_2_ can easily cross the barriers of the brain and will activate chemoreceptors leading to an increased exhalation of CO_2_.
- The low pH, however, is harmful and bases such as HCO_3_^−^ are imported across the brain barriers in order to normalize brain pH.
- We show that the HCO_3_^−^ transporter NBCe2 in the choroid plexus of the blood‐cerebrospinal fluid barrier is absolutely necessary for normalizing CSF pH during high levels of CO_2_.
- This discovery represents a significant step in understanding the molecular mechanisms behind regulation of CSF pH during acid‐base disturbances, such as chronic lung disease.

## Introduction

Normal neuronal function within the central nervous system (CNS) relies on a stable and suitable internal physico‐chemical environment, where interstitial pH is amongst the important parameters dictating neuronal excitability (Leusen, 1972; Hladky & Barrand, 2016). Disturbances of brain pH affect neuronal function due to altered protonization of the proteins in the membranes governing the electrical properties of the cells (Somjen, 1984), such that severe acidosis results in confusion, coma, and ultimately death (Posner & Plum, 1967), whilst brain alkalosis leads to convulsions such as febrile seizures (Schuchmann *et al*. 2006). The main regulatory systems to correct acid‐base disturbances are changes in the pulmonary ventilation (controlling $P_{CO_{2}}$) and the renal excretion of acid‐base equivalents (i.e. net NH_4_^+^, H^+^ or HCO_3_^−^ secretion) (Siesjö, 1972). The ventilatory response depends primarily on central chemoreceptors sensing $P_{CO_{2}}$ and H^+^ within the brain interstitial fluid (Kazemi & Johnson, 1986).

Both the blood‐brain and the blood‐CSF barriers are highly permeable to CO_2_, but much less so to H^+^ and HCO_3_^−^ (Johnson *et al*. 1983). Increases in arterial $P_{CO_{2}}$ are therefore quickly sensed by the central chemoreceptors enabling swift respiratory responses to hypercapnia. Numerous classic studies, nevertheless, also demonstrate that acute changes in blood pH during metabolic acid‐base disturbances are conveyed to the CSF (Pappenheimer *et al*. 1965; Kazemi *et al*. 1967; Yuan & Desiderio, 2005). Thus, although CSF $P_{CO_{2}}$ quickly follows plasma $P_{CO_{2}}$, transepithelial ion transport across the blood‐brain and the blood‐CSF barriers enables [HCO_3_^−^] within the CSF to counteract the changes in $P_{CO_{2}}$ and hence restore CSF pH even in the absence of changes in plasma [HCO_3_^−^] (reviewed in (Siesjö, 1972)). In dogs, Kazemi and co‐workers showed that CSF pH is normalized within 6 h of 10% CO_2_ inhalation despite low plasma pH, indicating that CSF, despite its low content of protein buffers, is efficiently protected against acid‐base disturbances by either removal of acid or import of base equivalents, such as HCO_3_^−^ into the CSF (Kazemi *et al*. 1967). The choroid plexus epithelium (CPE) is suggested to mediate the blood‐to‐CSF transport of H^+^ and HCO_3_^−^ in the response to acid‐base disturbances. It does so by either transporting e.g. HCO_3_^−^ from the blood to CSF or by *de novo* synthesis of HCO_3_^−^ for extrusion to the CSF (Hasan & Kazemi, 1976). The active extrusion of HCO_3_^−^ into the CSF by the blood‐CSF barrier, i.e. the CPE, was first suggested as a compensatory mechanism in respiratory acidosis by Maren (1971). Although there are clear indications for a role in CSF pH regulation by the CPE, the underlying molecular mechanisms of this phenomenon remains elusive.

The CPEs reside in each of the four brain ventricles, and this very active epithelial monolayer is the primary source of intraventricular CSF (Damkier *et al*. 2013). The CPE cells (CPECs) contain a number of membrane transport proteins involved in secretion of electrolytes and water, and express a variety of other proteins involved in movement of acid‐base equivalents across the plasma membrane (Damkier *et al*. 2013). Among the transporters expressed in the luminal (CSF‐facing) plasma membrane, the electrogenic Na^+^‐HCO_3_^−^ cotransporter NBCe2 is the only known base extruder (Bouzinova *et al*. 2005; Millar & Brown, 2008; Damkier *et al*. 2013). NBCe2 is known to export Na^+^ and HCO_3_^−^ from the epithelial cell to the CSF with a 1:3 stoichiometry (Millar & Brown, 2008). The localization and transport direction of NBCe2 makes it an obvious candidate for maintenance of CSF pH during acidosis in the brain. In the basolateral membrane, the Na^+^ dependent Cl^−^/HCO_3_^−^ exchanger Ncbe (*Slc4a10*) imports Na^+^ and HCO_3_^−^ from the blood side (Praetorius *et al*. 2004). The anion exchanger AE2 is also expressed in the basolateral membrane where it is responsible for extrusion of HCO_3_^−^ from the cell (Alper *et al*. 1994). The electroneutral Na^+^‐HCO_3_^−^ cotransporter, NBCn1 is also expressed in the CPE (Bouzinova *et al*. 2005), but the membrane localization of this transporter varies between species (Praetorius *et al*. 2004). It transports Na^+^ along with HCO_3_^−^ into the cell. In the luminal membrane the Na^+^/H^+^ exchanger NHE1 extrudes H^+^ from the cell in exchange for Na^+^ (Damkier *et al*. 2009). The contribution of these transporters to CSF pH regulation remains to be quantified.

Two previous studies have investigated the consequences of genetically deleting NBCe2 in mice (Kao *et al*. 2011; Gröger *et al*. 2012). In the study by Kao *et al*. (2011), *Slc4a5* deletion was accomplished by insertion of a gene trap vector, which integrated upstream of exon 15. In the study by Gröger *et al*. (2012), *Slc4a5* was deleted by insertion of loxP sites targeting exon 7. In both cases, a frameshift mutation and following truncation of the final NBCe2 protein resulted in deletion of NBCe2. The effects resulting from *Slc4a5* deletion differed between the two models. In the study by Kao *et al*., immunohistochemical analysis revealed disrupted expression of the electroneutral Na^+^‐HCO_3_^−^ cotransporter Ncbe (*Slc4a10*). This protein is normally exclusively expressed in the basolateral membrane, but in the NBCe2 knockout (KO) Ncbe was expressed in both membranes. In addition, striking changes in subunit expression of the Na^+^,K^+^‐ATPase were observed: the α1 subunit was found in the basolateral membrane as well as in the luminal membrane of CPECs in the NBCe2 KO mouse. The β2 subunit was absent from the NBCe2 KO CPE. Expression of the luminal Na^+^‐K^+^‐2Cl^−^ cotransporter, NKCC1, and the cytoskeletal protein spectrin βII was observed both intracellularly and luminally in the knockout mice. Most of these proteins are involved in CSF secretion by the CPE (Damkier *et al*. 2013), and indeed brain ventricle volume and intracranial pressure were dramatically decreased in this study. Electrolyte analysis showed that the NBCe2 KO mice had reduced CSF [HCO_3_^−^] indicating a deficiency in acid‐base regulation of the CSF. Injections with the convulsant pentylenetetrazol (PTZ) revealed that the NBCe2 KO mice had lower susceptibility to chemically induced seizures. In contrast to these findings, no difference in brain ventricle volume was detected by Gröger *et al*. (2012), indicating a less severe NBCe2 KO phenotype in this mouse model. The distribution of the CPE transporters and the electrolyte composition of the CSF was not investigated in this study. Nevertheless, the difference in ventricle phenotype suggests a profound difference between the two knockout models, which could be ascribed to differences in gene targeting strategies. Both techniques gave rise to verified frame shifts and truncation of the resulting proteins. The gene trap insertion results in the expression of a relatively large non‐functional NBCe2 protein including the first transmembrane domains that potentially could interfere with the expression of other CPE proteins. The exon 7 deletion approach might carry the risk of alternative spliced NBCe2 forms being expressed, but mass spectrometer analysis of the NBCe2 KO seems to rule this out. Without knowing the exact reason for the discrepancies and which knockout model most likely reflects NBCe2 function, a novel mouse model of NBCe2 deletion, in which the potential for truncation and splice variants can be avoided, seems warranted.

In the present study, we generated a knockout model targeting the conserved first transmembrane segments of NBCe2. This prevents signal peptide‐mediated transfer into the rough endoplasmic reticulum (RER). The insertion of truncated protein into the membrane was hindered, as validated using a novel antibody directed at the N‐terminal of NBCe2. We hypothesize that HCO_3_^−^ transport via NBCe2 in the CPE is the main molecular mechanism to modulate CSF pH in face of acute respiratory acidosis. We exploit a novel NBCe2 knockout mouse generated using the Cre‐Lox system, as well as a targeted siRNA NBCe2 knockdown (KD) approach to investigate the role of NBCe2 in the regulation and maintenance of CPE and CSF pH. We show that knockout of NBCe2 reduces the base extrusion rate in CPECs during recovery from intracellular alkalization. *In vivo* intraventricular recordings of CSF pH in NBCe2 KO and NBCe2 KD mice demonstrate that NBCe2 is necessary for sustaining CSF pH recovery from hypercapnia‐induced acidification. Thus, we suggest HCO_3_^−^ extrusion through NBCe2 as the first mechanistic insight into local compensatory regulation of CSF pH by the CPE. Knowledge of the molecular mechanisms involved in CSF alkalization as well as their regulation may prove beneficial in conditions where a pharmacological approach to adjust CSF pH is clinically desirable, for example during seizures or acid‐base disturbances.

## Methods

### Ethical approval

All animal experiments conform to the national guide for the care and use of laboratory animals and all experimental protocols were approved by the national authority, The Danish Animal Experiments Inspectorate. Experiments were conducted in C57BL/6 mice (Taconic Biosciences, Ejby, Denmark). Unless otherwise stated, only male mice aged 8–12 weeks were used. Mice were fed a rodent pellet diet (Altromin 1319, Brogaarden, Lynge, Denmark) *ad libitum*, had free access to tap water and were housed in a temperature‐controlled room with a 12 h:12 h light‐dark cycle. All mice were killed by cervical dislocation following the experiments. The investigators conform to the ethical principles and animal checklist required by *The Journal of Physiology*.

### Generation of global *Slc4a5* knockout (NBCe2 KO)

**Figure 1:**
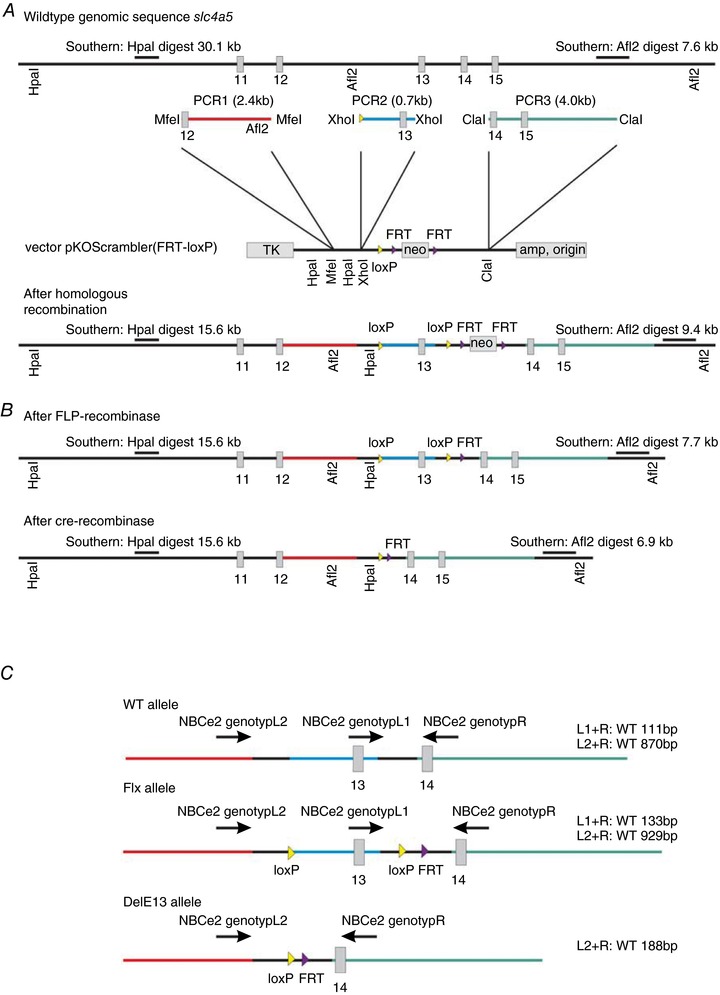
Generation of NBCe2 (*Slc4a5*) floxed and knockout (KO) mice *A*, schematic drawing depicting the targeting strategy used for generation of mice with a ‘floxed’ NBCe2 gene. The exons 11 to 15 are indicated on the *Slc4a5* wild‐type (WT) genomic sequence and PCR products. The positions of the Southern probes, PCR primers, and vector restriction sites used in generation of the targeting construct and for screening of the ES cell clones are indicated. *B*, similar representation of the floxed gene after removal of the neo cassette by FLP recombination (top) and the exon 13‐deleted gene after Cre‐recombination (bottom). *C*, schematic drawing of the annealing sites of the PCR primers used in genotyping, and the expected product sizes of genotyping for the wild‐type, floxed and deleted alleles, as indicated.

The targeting construct for creating the ‘floxed’ *Slc4a5* gene encoding NBCe2, with loxP sites flanking exon 13, was generated by insertion of PCR‐amplified genomic DNA segments spanning the *Slc4a5* gene sequence into a modified pkoScrambler (FRT‐loxP) vector (Table 1, Fig. 1 *A*). The targeting construct was linearized and electroporated into CJ7 embryonic stem (ES) cells derived from 129S1/Sv mice (Swiatek *et al*. 1994). G418‐resistant colonies were selected and expanded. The clones with homologous recombination were identified by Southern blot with probes flanking the targeting construct sequence. To generate the conditional floxed *Slc4a5* ^TMIEmfu^ allele, the neomycin phosphotransferase expression cassette, which was flanked by FRT sites, was deleted by transient transfection of targeted ES cells with a FLP‐recombinase expression plasmid (Fig. 1 *B*). The neomycin‐sensitive clones were validated by Southern blotting with a probe generated by amplification of a genomic fragment using screening primers (Table 1). In following sections this is called the *Slc4a5* ^flx^ allele.

**Table 1:** Primers for Southern blot, genotyping, and RT‐PCR

Primers	Sequence	Product size
Amplification of genomic fragment 1	cctCAATTGCAGAGCCGGGCCAGATGAAT gggCAATTGACAGTCATTTGGGAGATGGGTCTCT	2380 bp
Amplification of genomic fragment 2	cccCTCGAGATAACTTCGTATAGCATACATTATACG‐AAGTTATGACAGTTCCCACTAACCATTTCAT gggCTCGAGTGATTTCCCTAGAAGTCCAGCCTA	697 bp
Amplification of genomic fragment 3	ccaATCGATGGCTAATTGTGACCTCCCTACATT ccaATCGATAGCGCCTGTGGTAAGACCTCTTTAG	3956 bp
Probes for screening of ES clones	L: GTGAGTCTTCTCGACGGCAAATCTT R: GAAAAGGAGAGTGTCCCTAGCAAGC	813 bp
Mouse genotyping	L1: AGGCTGGACTTCTAGGGAAATCAC L2: TTCCCAATCAATCCACAAAGTCAAG R: AATGTAGGGAGGTCACAATTAGCCA	WT 111 bp FLX 133 bp KO 188 bp

Chimeric mice were generated by injection of the ES cells into B6D2F2 mouse blastocysts (Wertz & Füchtbauer, 1994). Chimeric males were bred with C57BL/6 females, and agouti offspring (indicating germ‐line transmission of the manipulated 129S1/Sv ES cells) were analysed for the presence of the *Slc4a5* ^flx^ mutation by PCR using genomic tail DNA and the primers L1 and R (Table 1). The expected product for the wild‐type (WT) allele was 111 bp and 133 bp for the *Slc4a5* ^flx^ allele.

The *Slc4a5* ^TM1.1Emfu^ KO allele was obtained by breeding mice carrying the *Slc4a5* ^flx^ allele with a tamoxifen‐inducible ubiquitin promoter‐driven Cre‐recombinase expressing mouse strain, B6.Cg‐Tg(UBC‐cre/ERT2)1Ejb/1J (The Jackson Laboratory, Scanbur, Karlslunde, Denmark). The resulting mice were treated with intraperitoneal injections of tamoxifen in sunflower seed oil to induce Cre‐recombinase expression and the female mice were subsequently used for breeding full NBCe2 knockout, heterozygous NBCe2 (HZ), and wild‐type mice The deleted *Slc4a5* ^TM1.1Emfu^ allele lacking exon 13 is in the following called *Slc4a5* ^delE13^. *Slc4a5* ^delE13^was detected as a 188 bp PCR product using primer pair L2 and R (Table 1, Fig. 1 *C*). Thus, all mice used in the experiments were on a mixed C57Bl/6J‐129S1/Sv genetic background, and therefore littermates are compared with NBCe2 KO mice throughout the study.

### Genotyping

The genotypes of all littermates were determined by polymerase chain reaction (PCR) of genomic DNA from tail biopsies. Tails were boiled at 95°C for 30 min in 25 mm NaOH and 0.2 mm EDTA and then an equal volume of 40 mm Tris‐HCl was added. For the PCR reaction, a total of 20% DNA‐containing solution was mixed with 5 pmol of each primer (Table 1) and 5× FIREPol Blend Master Mix (Solis BioDyne, Tartu, Estonia). After activation at 95°C for 5 min, PCR was performed for 30 cycles: Denaturation at 95°C for 30 s, annealing at 58°C for 30 s, and elongation at 72°C for 1 min. PCR products were visualized with DNA gel loading dye (6×, Thermo Scientific, Waltham, MA, USA) containing 0.05% 10000× GelRed nucleic acid gel stain (Biotium, Fremont, CA, USA).

### Anti‐NBCe2 antibodies

A 16 amino acid peptide with an N‐terminal cysteine (CMNDISHTPNTDQRKNK) corresponding to amino acid residues 162 to 177 in the N‐terminal domain of mouse NBCe2 (*Slc4a5*, NP_001159539.1) was used for immunization of rabbits (Genscript, Piscataway, NJ, USA) and yielded an antiserum titre higher than 1:512,000 (i.e. maximal sample/blank ELISA ratio at A450nm). The antibody was affinity purified with the immunizing peptide coupled to an agarose column (SulfoLink, Thermo Fisher Scientific, Fremont, CA, USA).

The antibodies were validated by immunocytochemistry of cell cultures expressing NBCe2. Flp‐In‐3T3 cells (Invitrogen, Carlsbad, CA, USA) were transfected with wild‐type NBCe2 (Genscript) in a pcDNA™5/FRT (Invitrogen) vector and selected with hygromycin B. Cells were grown in Dulbecco's modified Eagle's medium supplemented with donor bovine serum (10%), at 37°C with 5% CO_2_.

NBCe2‐transfected cells were washed with a phosphate‐buffered salt solution (PBS, in mm: 167 Na^+^, 2.8 H_2_PO_4_^−^, 7.2 HPO_4_^2−^, pH 7.4) and immersion fixed with 4% paraformaldehyde. The cells were permeabilized in 0.2% saponin for 10 min, and excess binding sites were blocked with a serum solution (10% fetal calf serum, 0.1% BSA, 0.05% saponin) and a gelatin solution (0.2% fish gelatin, 1% BSA, 0.05% saponin, 0.05 m glycine). The cells were then incubated with the primary antibody over night at 4°C. A goat anti‐rabbit Alexa Fluor 488 antibody (Invitrogen) was used for visualization.

### Immunoblotting

Choroid plexus was dissected from the brain of NBCe2 KO and WT mice and transferred to sample buffer (0.3 m sucrose, 25 mm imidazole, 1 mm ethylenediaminetetraacetic acid, 0.1 m sodium dodecyl sulphate, and 0.04 m dithiothreitol, Bromophenol Blue, pH 6.8). Samples were sonicated by 5 bursts 3 times at 60% using a Model 150 V/T sonicator (BioLogics Inc., Cary, NC, USA) and heated for 15 min at 65°C. Samples were loaded on 4–12% polyacrylamide SDS gels and separated by electrophoresis. After transfer to a polyvinylidene difluoride membrane (PVDF, Ambion, Foster City, CA, USA), the membrane was blocked with 5% skimmed milk in PBS‐T (PBS with 0.1% vol/vol Tween). The membrane was incubated with the primary antibody in 1% BSA, 2 mm NaN_3_ in PBS‐T overnight at 4°C. After washing, the membrane was incubated with secondary antibody (goat anti‐rabbit HRP, 1:3000, Dako, Glostrup, Denmark) for 1 h at room temperature. ECL Plus (GE Healthcare) was used for visualization of immunoreactive bands using an ImageQuant LAS4000 (GE Healthcare, Chicago, IL, USA) chemiluminescence digital analyser.

### Immunohistochemistry

Mice were perfusion fixed via the heart with 4% paraformaldehyde in PBS. After fixation, the brain was post‐fixed for 2 h, dehydrated, and embedded in paraffin wax, which enabled 2 μm sectioning using a rotary microtome (Leica, Wetzlar, Germany). The sections were de‐waxed and stepwise rehydrated before epitopes were retrieved by boiling the sections in 10 mm Tris buffer (pH 9) with 0.5 mm EGTA. Aldehydes were quenched with 50 mm NH_4_Cl in PBS and unspecific binding was blocked by washing with 1% BSA in PBS with 0.2% gelatin and 0.05% saponin. Sections were incubated overnight at 4°C with the primary antibody diluted in 0.1% BSA in PBS added 0.3% Triton X‐100. Primary antibodies are listed in Table 2. Positive control tissues included kidneys, brain, vasculature, and red blood cells (not shown).

**Table 2:** Primary antibodies

Target	Antibody	Host	Reference
Na^+^,K^+^‐ATPase α1	3B‐0/56‐0	Mouse	Gift from Forbush (Kashgarian *et al*. 1985)
Na^+^,K^+^‐ATPase β1	SpET β1	Rabbit	Gift from Martín‐Vasallo (Gonzalez‐Martinez *et al*. 1994)
AQP1	2353 AP	Rabbit	Praetorius, similar to Terris (Terris *et al*. 1996)
NKCC1	C‐terminal	Rabbit	Gift from Turner (Kurihara *et al*. 1999)
AE2	C‐terminal	Rabbit	Gift from Stuart‐Tilley (Stuart‐Tilley *et al*. 1994)
NBCe	1139 AP	Rabbit	Praetorius (Praetorius *et al*. 2004)
NBCn1	ntNBCn1	Rabbit	Praetorius (Damkier *et al*. 2007)
αI‐Spectrin	LS‐C137722	Rabbit	LifeSpan, Seattle, WA, USA
αII‐Spectrin	sc‐46696 (C‐11)	Mouse	Santa Cruz Biotech, Dallas, TX, USA
βI‐Spectrin	LS‐C138700	Rabbit	LifeSpan
βII‐Spectrin	sc‐28272 (H‐125)	Rabbit	Novus, Biologicals. Littleton, CO, USA

The fluorescence visualization of the primary antibodies was performed using AlexaFlour 488‐ or 555‐coupled donkey anti‐goat, ‐sheep, ‐rabbit, or ‐mouse secondary antibodies (Invitrogen). Cell nuclei were visualized using Topro3 counterstaining (Invitrogen). Sections were mounted with a coverslip in Glycergel antifade medium (Dako) and analysed using a Leica DMIRE2 inverted microscope with a TC5 SPZ confocal unit using a 63×/1.32 NA objective. Semiquantitative analysis of immunofluorescence images were performed as described previously (Christensen *et al*. 2013).

### Intracellular pH recording by live cell microscopy

Isolated CP tissues were digested into single‐layered cell clusters by 4 μg ml^−1^ dispase (Invitrogen) and 4 μg ml^−1^ collagenase B (Roche, Penzberg, Germany) in calcium‐free HBS (Table 3) at 37°C for 30 min. The digested cell clusters were mounted on Cell‐Tak (BD Biosciences, Franklin Lakes, NJ, USA) coated coverslips for 10–15 min at 37°C and loaded for 10 min with the pH sensitive probe BCECF‐AM or carboxy‐SNARF (2 μm, Invitrogen). Coverslips were mounted in a closed perfusion chamber (RC‐21BR; Harvard Apparatus, Cambridge, MA, USA) and placed on an inverted microscope stage inside a 37°C dark box. Cells were allowed to equilibrate to a baseline level pH in HBS before the protocols were executed as detailed in the figures and legends.

**Table 3:** Salt solutions for live cell fluorescence microscopy

Substance (mM)	HBS	NH_4_Cl HBS	Na^+^‐free BBS	BBS	TMA BBS	Cl^−^‐free BBS	High K^+^ solution
Na^+^	145.0	125.0	0.0	145.0	135.0	145.0	10.0
K^+^	3.6	3.6	3.6	3.6	3.6	3.6	138.6
Ca^2+^	1.8	1.8	1.8	1.8	1.8	1.8	1.8
Mg^2+^	0.8	0.8	0.8	0.8	0.8	0.8	0.8
NH_4_^+^		20.0					
Cl^−^	138.6	138.6	138.6	138.6	138.6	0	138.6
SO_4_^−^	0.8	0.8	0.8	0.8	0.8	0.8	0.8
HCO_3_^−^			24.0	24.0	24.0	24.0	
Glucose	5.5	5.5	5.5	5.5	5.5	5.5	5.5
HEPES	10.0	10.0	10.0	10.0	10.0	10.0	10.0
NMDG			121.0				
Choline			24.0				
PO_4_^3−^	2.0	2.0	2.0	2.0	2.0	2.0	2.0
TMA					20.0		
mOsm	308	308	308	308	308	308	308
pH	7.40	7.40	7.40	7.40	7.40	7.40	7.00
CO_2_			5%	5%	5%	5%	

HBS, HEPES‐buffered solution; BBS, CO_2_/HCO_3_^−^‐buffered solution; HEPES, (4‐(2‐hydroxyethyl)‐1‐piperazineethanesulfonic acid; TMA, trimethylamine; NMDG: *N*‐methyl‐D‐glucamine.

For pH_i_ recording using BCECF, the cells were imaged at the stage of a Nikon Eclipse microscope equipped with a Nikon Plan Apo VC 60×/1.40 NA oil‐immersion objective. Till Vision software (Till Photonics, Martinsried, Germany) was used to control monochromator wavelength alternating between 490 nm and 440 nm, exposure time (20 ms), frequency (1 Hz), and binning (to 640 × 480 pixel images). The light emission at 510–535 nm was recorded by a 12‐bit cooled monochrome CCD camera (Imago, Till Photonics) and data were collected from user‐defined regions of interest (ROIs) of individual cells after background subtraction. Sample size (*n*) refers to the mean values from at least three individual CP cells from one mouse. In separate experiments the excitation fluorescence ratio (490/440 nm) was calibrated to pH by clamping pH_i_ stepwise from pH 8 to 6 in high‐K^+^ HBS with 10 μm nigericin (Boyarsky *et al*. 1988; Damkier *et al*. 2010). Each experiment was concluded with a one‐point calibration in pH 7.0, high‐K^+^ HBS with 10 μm nigericin (Table 3).

For pH_i_ recording using SNARF, the cells were imaged using an iMic microscope (Till Photonics) with an Olympus UApo N340, 40×/1.35 NA oil‐immersion objective. Till Vision software (Till Photonics) was used to control monochromator wavelength for excitation alternating between 485 nm and 555 nm, exposure time (25 ms), frequency (0.25 Hz), and binning (to 256 × 256 pixel images). The light emission at 565–615 nm was recorded by a 14‐bit cooled monochrome EMCCD camera (iXon^EM+^, Andor Technology, Belfast, UK) with 4× EM gain, and data were collected from user‐defined regions of interest (ROIs) of individual cells after background subtraction. The excitation fluorescence ratio (485/555 nm) was calibrated to pH by clamping pH_i_ stepwise from pH 8.4 to 7 in high‐K^+^ HBS with 10 μm nigericin. Each experiment was concluded with a one‐point calibration in pH 7.5, high‐K^+^ HBS with 10 μm nigericin (Table 3).

The rate of pH_i_ recovery (dpH_i_/d*t*) was determined as the pH_i_ change in 30 s after peak or nadir pH_i_. The net acid or base efflux was calculated as the product of the total buffering capacity (βtot) and the dpH_i_/d*t*. The βtot was calculated as the sum of the intrinsic buffering capacity (βint) and the contribution of the CO_2_/HCO_3_^−^ buffering system (Boyarsky *et al*. 1988). The intrinsic buffering capacity was determined by recording pH_i_ changes during stepwise decreasing NH_4_^+^ concentrations from 20 to 0 mm as previously described (Damkier *et al*. 2010).

### Barometric measurements

Ventilatory responses to 5% CO_2_ were measured using the barometric method as previously described (Iversen *et al*. 2012). Awake, unrestrained mice were placed individually in a closed thermostated chamber (1.1 l) and allowed to acclimatize overnight. Room air was pumped (EHEIM 400, Deizisau, Germany) through the chamber at a rate of approximately 300 ml min^−1^. The excurrent flow from the chamber was connected to a gas analysing system measuring the fractional concentrations of O_2_ and CO_2_ (Ametek, Applied Electrochemistry, CD‐3A & S‐3A, Berwyn, PA, USA). A humidity sensor (Humitter 50Y, Vaisala, Vantaa, Finland) measured the relative humidity inside the chamber and a differential pressure transducer (First Sensor HCLA02x5DB, Berlin, Germany) measured the ventilation driven pressure. After approximately 16 h the chamber was closed and ventilatory frequency and tidal volume were measured at baseline conditions (room air) for 5 min. The chamber was then flushed for 15 min and closed again. Now CO_2_ was injected into the chamber to a final concentration of 5% and the recording of ventilator frequency and tidal volume was repeated. Volume‐related pressure signals were collected by a BIOPAC MP 100 data acquisition system at a sample rate of 200 Hz. Volume calibration was performed by injecting and withdrawing known volumes with a calibrated glass syringe. At the end of each experiment, body temperature was measured using a rectal thermocouple. Ventilatory pressure traces were exported to Mathematica (Version 7.0, Wolfram Research) followed by analysis in a script that detected all peaks and calculated their amplitude and frequency. From these analyses the tidal volume (*V*_T_) for each animal could be calculated from the equation given by Drorbaugh & Fenn (1955):

$$V_{T}=V_{K}\frac{P}{P_{K}}\frac{T_{A}(P_{B}-P_{C})}{T_{A}(P_{B}-P_{C})-T_{C}(P_{B}-P_{A})}.$$

*V*_K_ is the volume of the glass syringe used for calibration, *P* is the amplitude of the pressure trace, *P*_K_ is the amplitude resulting from the volume injected with the glass syringe, *T*_A_ is the body temperature of the mouse, *P*_B_ is the barometric pressure of the day (read prior to each experiment), *P*_C_ is the water pressure inside the chamber, *T*_C_ is the chamber temperature, and *P*_A_ is the saturated water pressure inside the lungs of the mouse.

### Blood gas analysis

Mixed arterial and venous blood samples were drawn from the right heart atrium of isoflurane‐anaesthetized mice using heparin‐containing PICO syringes (Radiometer, Bronshoj, Denmark) and blood gas was analysed on an ABL80 FLEX blood gas analyser (Radiometer).

### Generation of brain ventricle *Slc4a5* knockdown (NBCe2 KD) mice

Wild‐type C57BL/6 mice (Taconic) were anaesthetized using intraperitoneal injections of ketamine (100 mg kg^−1^, Ketaminol, MSD Animal Health, Copenhagen, Denmark) and xylazine (10 mg kg^−1^, Xysol vet, ScanVet Animal Health A/S, Fredensborg, Denmark) in saline. When adequately anaesthetized, the mouse was mounted in a stereotaxic device (David Kopf Instruments, Tujunga, CA, USA), and a microlitre Hamilton syringe (ILS Innovative Labor Systeme GmbH, Stützerbach, Germany) containing endoribonuclease‐prepared siRNA pools (MISSION esiRNA, Sigma‐Aldrich, St. Louis, MO, USA) targeting *RLuc* (Renilla luciferase, control) or mouse *Slc4a5* was placed in the lateral ventricle. The optimal stereotaxic coordinates for the needle placement were established by injecting Fast Green dye, and were set to 0.1 mm posterior, 0.8 mm lateral, 2.5 mm ventral. The brain tissue was allowed to seal around the needle for 3 min, after which 10 μl of 200 ng μl^−1^ siRNA was delivered into the cerebroventricular system at a rate of 0.5 μl min^−1^. After the injection, the needle was left inside the brain for 5 min to prevent backflow of CSF and siRNA. Upon removal of the needle, the incision was sutured and the mouse was allowed to recover under a heating lamp. A dose of 0.05 mg kg^−1^ Buprenorphine (Buprenodale vet, Lostock Gralam, UK) was administered subcutaneously for analgesia.

### *In vivo* cerebrospinal fluid pH measurements in NBCe2 KO, KD and WT mice

The *in vivo* cerebrospinal fluid pH measurements were performed on anaesthetized mice by placing a pH electrode in the lateral ventricle (as described above). Two types of pH electrodes were tested: First, a glass micro‐electrode with a 1.1 mm diameter protective needle (PH‐N, Unisense, Aarhus, Denmark) was calibrated in pH 4, 7, and 10 buffers (VWR chemicals, Radnor, PA, USA) and placed in the right lateral ventricle. Then a small (4 × 4 mm) hole was drilled on the left side of the skull with an Ideal Micromotor drill (CellPoint Scientific, Gaithersburg, MD, USA) and the reference electrode (REF‐100, Unisense) was slowly lowered until it touched the brain tissue. The pH signal was measured every 3 s with a pH/mV‐Meter (Unisense) and analysed with SensorTrace Logger software (Unisense). Second, a needle‐type optical pH microsensor (NTH‐HP5, 140 μm needle tip diameter) connected to a pH‐1 micro transmitter (PreSens GmbH, Regensburg, Germany) was calibrated by a multipoint calibration in pH 4, 6, 7 and 8 buffers at 22°C (for proof of concept and baseline pH measurements) or by a one‐point calibration in a pH 7 buffer at 37°C (for pH recovery measurements). The microsensor was inserted into the right lateral ventricle and pH was measured every 2 s. A rectal temperature probe connected to the pH transmitter was used to compensate for temperature‐dependent pH changes. For proof of concept experiments, both electrodes were tested by placing a 10 μl Hamilton syringe (ILS Innovative Labor Systeme GmbH) parallel to the pH electrode in the left lateral ventricle. After determining the baseline pH value, 1 μl of 5 mm HCl was injected and the changes in pH were continuously monitored for 5 min. Between the two electrodes, the chemical optical pH microsensor proved to be more suitable for our purposes and was used in the following experiments.

Baseline CSF pH values were determined during a 5 min period after stabilization of the pH electrode. In the hyperapnoea experiments a gas mixture containing 5% CO_2_ in normal air (AGA, Pullach, Germany) was administered via a nosepiece attached to the stereotaxic device. Mice were allowed to inhale the 5% CO_2_ gas mixture for a 30 min period, after which they were switched back to inhaling normal room air. The mice were kept anaesthetized by administration of small doses of the ketamine/xylazine mixture described above for the duration of the experiments. In the subsequent analysis, CSF pH values during the last 20 min of CO_2_ exposure were compared. Calculations were based on averaged pH/minute. The measurements were performed on the NBCe2 KO and WT mice, as well as on the NBCe2 KD mice in which baseline pH was determined 24 and 48 h after injecting siRNA, whereas the recovery was measured 48 h after siRNA injection. All mice were killed following the experiments.

### qPCR

The choroid plexus was rapidly dissected and placed into RNAlater Stabilization Solution (Ambion). Total RNA was purified using GeneJet RNA purification kit (Thermo Fisher Scientific). The concentration of purified RNA was determined by absorbance at 260 nm using a NanoDrop ND‐2000 (Fisher Scientific). Then 80–120 ng of RNA was reverse transcribed using iScript Reverse Transcription Supermix (BioRad). qPCR amplification was performed with Step One Plus real time PCR system (Applied Biosystems, Foster City, CA, USA) using a commercially available TaqMan Gene Expression Assay (*Slc4a5*: Mm01190997_m1, control – *Actb*: Mm00607939_s1; Applied Biosystems) and the universal TaqMan Gene Expression Master Mix. PCR cycling conditions were 50°C for 2 min, 95°C for 10 min, followed by 40 cycles of 95°C for 15 s and 60°C for 1 min. The results are presented as the relative NBCe2 expression fold‐change (2^−ΔΔCT^ method) compared to the calibrator: the *RLuc* siRNA‐injected mice.

### Seizure induction by pentylenetetrazol administration or hyperthermia

For pharmacological induction of seizures, mice were inhalation‐anaesthetized with isoflurane for 2 min and given a 45 mg kg^−1^ intraperitoneal injection of pentylenetetrazol (PTZ; Sigma). The mice were then placed in separate cages and monitored by video recording for 60 min. Seizure activity was analysed similar to Kao *et al*. (2011). Briefly, seizures were classified as follows: stage 0, no response; stage 1, facial twitching; stage 2, myoclonic jerks without upright position; stage 3, myoclonic jerks and upright position with bilateral forelimb clonus; stage 4, clonic‐tonic seizures; stage 5, generalized clonic‐tonic seizures with loss of postural control. All mice were killed when stage 5 was reached or after 60 min.

Hyperthermia‐induced seizures were induced by placing a heating lamp above a transparent cylindrical container and heating the air inside the container to 42^o^C similar to Christensen *et al*. (2017 *)*. The mice were placed in the container to cause hyperventilation and thereby reduce $P_{CO_{2}}$. The time from the mouse being placed in the heated container until development of seizures was determined for each genotype. The maximum time the mice spend in the container was 10 min. Following the experiment the mice were killed.

### Statistical analysis

Live cell imaging, blood gas, seizure data, and CSF pH measurements were analysed by Student's unpaired two tailed *t* test comparing NBCe2 KO or KD mice to wild type. Barometric data was analysed by two‐way ANOVA comparing the two independent variables: genotype (NBCe2 knockout *versus* wild type) and treatment (normal air *versus* 5% CO_2_). The ANOVA was followed by Sidak's multiple comparisons test. A *P* value <0.05 was considered statistically significant. qPCR data was analysed by unpaired two tailed *t* test and the error bars represent RQ_min_ and RQ_max_ values, which were defined by the standard error of the ΔC_T_ and the 95% confidence interval.

## Results

### Generation and validation of full NBCe2 KO mice

**Figure 2:**
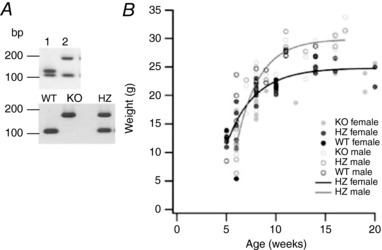
Basic characterization of the NBCe2 KO mouse line *A*, examples of genotyping of mice in the process of establishing full NBCe2 KO mice by crossing mice with floxed NBCe2 alleles with tamoxifen‐inducible ubiquitin‐promoter driven Cre‐expressing mice. In the top panel, the WT allele product of 111 bp is found in both mice, the floxed (FLX) allele product of 133 bp is found in mouse 1 (i.e. floxed NBCe2 on one allele), while the KO allele of 188 bp is found in mouse 2 (heterozygous (HZ)). The bottom panel shows genotyping of the offspring of heterozygous NBCe2 breeding. The offspring were WT (111 bp), KO (188 bp), or FLX (133 bp), respectively. *B*, weight gain of the NBCe2 KO mouse line. The figure shows individual observations of weight for male and female mice of each genotype NBCe2 KO, HZ and WT.

Examples of genotyping in the process of generating NBCe2 KO mice are shown in Fig. 2 *A*. The top panel shows the identification of both WT/flx mice and WT/KO (i.e. HZ mice) as the offspring from crossing WT/flx males and tamoxifen‐treated WT/flx females. The bottom panel shows genotyping results from heterozygous breeding, which yields NBCe2 WT, KO and HZ mice. With 832 live births, the distribution among genotypes was: 203 WT, 403 HZ, and 226 KO mice, with a gender distribution of 441 females and 391 males. The development of body weight over time for male and female mice of all three genotypes is illustrated in Fig. 2 *B*. There are no obvious differences among genotypes, but the expected trend for male mice to gain weight faster than the female is observed. The only difference in body weight among genotypes was observed in male mice after 6 weeks (mean weight NBCe2 KO: 11.1 ± 0.6 g, *n* = 5; NBCe2 HZ: 10.9 ± 1.3 g, *n* = 6, and NBCe2 WT: 19.7 ± 2.3 g, *n* = 3, *P* < 0.05), where NBCe2 KO and HZ male mice were significantly smaller than NBCe2 WT mice. This difference was eliminated at week 11 and beyond.

**Figure 3:**
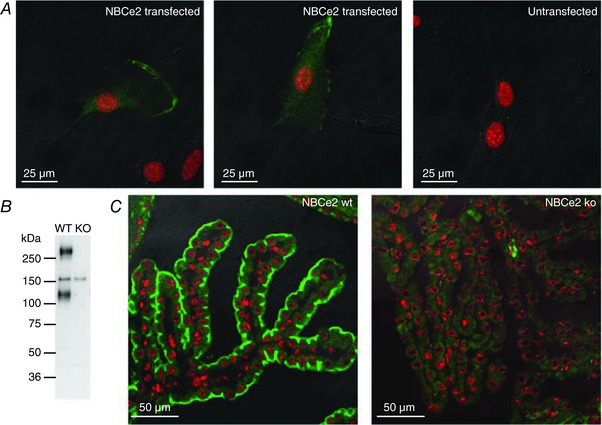
Validation of the NBCe2 KO mouse line *A*, representative anti‐NBCe2 immunostaining of NBCe2‐transfected and untransfected NIH‐3T3 cells, as indicated. NBCe2 immunofluorescence is shown in green, while nuclei are red. *B*, the same antibody was applied for immunoblotting choroid plexus proteins samples from NBCe2 WT and KO mice, as indicated. *C*, immunofluorescence staining of NBCe2 WT and KO mouse choroid plexus using the same anti‐NBCe2 antibody.

**Figure 4:**
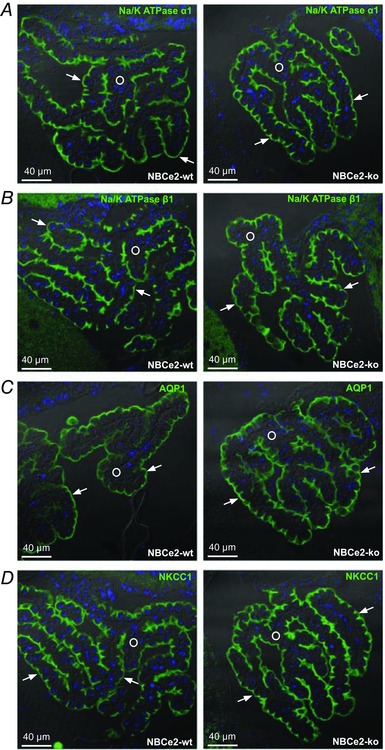
Localization of Na^+^,K^+^‐ATPase, AQP1 and NKCC1 in CPE from NBCe2 WT and KO mice The membrane localization of key transporters for CSF secretion by the CPE was studied on sections from NBCe2 WT and KO mouse brains subjected to immunofluorescence histochemistry. At least two mice of each genotype were analysed. *A*, representative images of the Na^+^,K^+^‐ATPase α1 subunit staining in NBCe2 WT and KO mouse brains, as indicated. *B*, similar staining for the Na^+^,K^+^‐ATPase β1 subunit. *C*, immunostaining to determine AQP1 localization in CPE from NBCe2 WT and KO mice. *D*, analysis of NKCC1 localization in the two indicated genotypes. In all micrographs, nuclei (blue) are stained with Topro3, arrows indicate the luminal membrane, and a circle is placed in the interstitial tissue.

**Figure 5:**
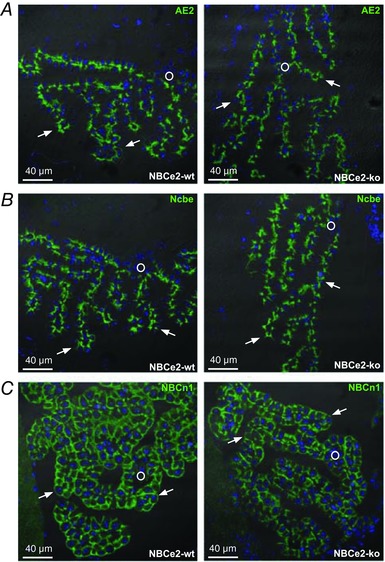
Localization of AE2, Ncbe and NBCn1 in CPE from NBCe2 WT and KO mice The membrane localization of bicarbonate transporters in the CPE was studied on sections from NBCe2 WT and KO mouse brains. At least two mice of each genotype were analysed. *A*, representative images of the AE2 staining in NBCe2 WT and KO mouse brains, as indicated. *B*, immunostaining to determine NBCe localization in CPE from NBCe2 WT and KO mice. *C*, analysis of NBCn1 localization in the two indicated genotypes. In all micrographs, nuclei (blue) are stained with Topro3, arrows indicate luminal membrane and a circle is placed in the interstitial tissue.

To validate the novel anti‐NBCe2 antibody, NBCe2‐transfected and untransfected NIH‐3T3 cells were immunostained. Figure 3 *A* shows that only cells transfected with NBCe2 produced immunoreactivity, confirming the sensitivity of the antibody. To confirm the knockout of NBCe2, isolated CP tissues from NBCe2 WT and KO mice were subjected to immunoblotting (Fig. 3 *B*). The anti‐NBCe2 antibody produced prominent bands of approximately 130 kDa and 260 kDa only in NBCe2 WT mice, which correspond to the expected molecular weight of the monomer and dimer forms of the protein, respectively. Immunohistochemical staining using the same antibody revealed luminal CPE membrane domain staining only in NBCe2 WT mice (Fig. 3 *C*). A previously published NBCe2 KO mouse revealed reorganization of key proteins involved in water and salt transport, as well as severe cytoskeletal rearrangements in the CPE (Kao *et al*. 2011). This reorganization was not confirmed in our model. The NBCe2 KO mice in our study display normal localization of the Na^+^,K^+^‐ATPase, the water channel aquaporin 1, AQP1, and the Na^+^‐K^+^‐2Cl^−^ cotransporter, NKCC1 (*Slc12a2*), in the luminal plasma membrane domain where they are normally found (Fig. 4). The bicarbonate transporters, the Cl^−^/HCO_3_^−^ exchanger AE2 (*Slc4a2*) and the Na^+^‐dependent Cl^−^/HCO_3_^−^ exchanger Ncbe (*Slc4a10*) showed normal localization in the basolateral membrane domain (Fig. 5 *A* and *B*). Interestingly, we found that the electroneutral Na^+^‐HCO_3_^−^ cotransporter NBCn1 (*Slc4a7*) is expressed both in the luminal and basolateral membrane. This localization pattern was, however, similar in NBCe2 WT and KO mouse CPE (Fig. 5 *C*). The localization of the α‐ and β‐spectrin isoforms was also investigated and showed similar distribution in both NBCe2 WT and KO mouse (not shown).

### The Na^+^‐dependent acid‐base transport in the CPE is affected by NBCe2 KO

**Figure 6:**
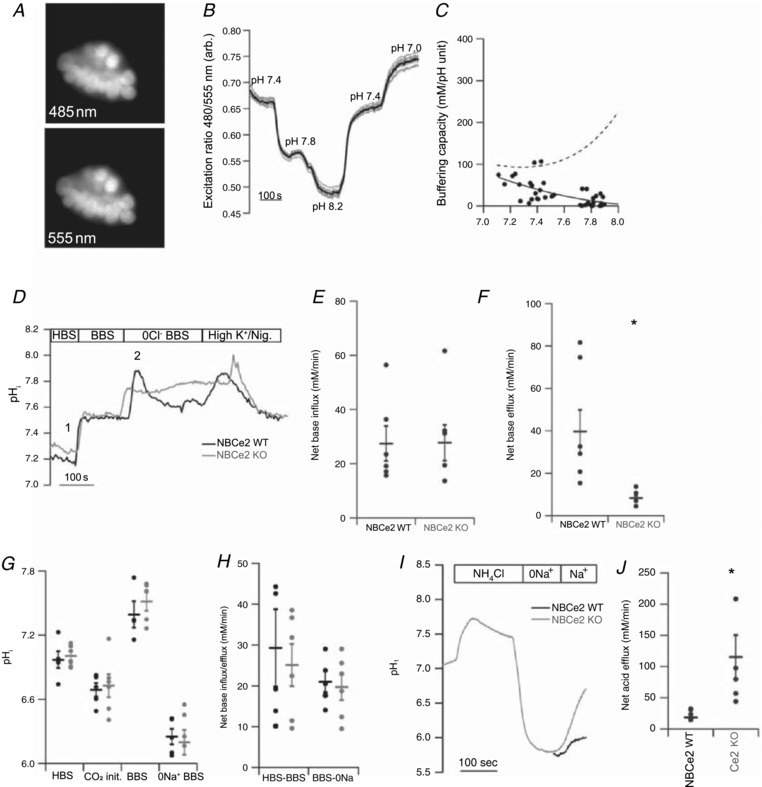
Na^+^‐dependent acid‐base transport in the CPE is affected by NBCe2 KO *A*, SNARF‐loaded isolated choroid plexus islets excited at 485 nm (top image) and 555 nm (bottom image) wavelength. *B*, traces of SNARF calibration experiments with excitation ratio shown as a function of time. Intracellular pH was clamped to extracellular pH as indicated by superfusing with a HEPES‐buffered solution containing a high [K^+^] and nigericin. The grey lines show all experimental traces from one experiment and the black line shows the mean values. *C*, plot of the measured intrinsic buffering capacity (continuous line) as well as the combined intrinsic buffering capacity and the theoretical contribution of the CO_2_/HCO_3_^−^ buffering system (total buffering, dashed line) at the corresponding level of pH_i_. *D*, representative traces of intracellular pH recordings in SNARF‐loaded isolated choroid plexus cells from NBCe2 KO (grey line) and WT (black line) mice. Baseline pH_i_ was determined in the absence (HBS) and presence (BBS) of CO_2_/HCO_3_^−^. Then the cells were alkalinized by removing Cl^−^ in the continued presence of CO_2_/HCO_3_^−^. The experiment was ended by a 1‐point calibration in a high‐K^+^‐nigericin solution with pH 7.5 (Nig). Points 1 and 2 refer to the pH changes calculated in *E* and *D*, respectively. *E*, mean values of the rate of pH_i_ increase (dpH/d*t*) mediated by the addition of CO_2_/HCO_3_^−^ (point 1 in panel *D*). *F*, mean values of the net base efflux ± SEM estimated at the peak alkalization after Cl^−^ removal (point 2 in panel *D*). *G*, mean values of intracellular pH_i_ measured by BCECF fluorometry in NBCe2 KO (grey dots) and wild‐type mice (black dots) at baseline in a HEPES‐buffered solution (HBS). The addition of CO_2_/HCO_3_^−^ induced a rapid CO_2_‐induced acidification (CO_2_ init.) followed by a new steady state pH_i_ mediated by the import of HCO_3_^−^ (BBS). Finally, Na^+^ was removed in the continued presence of CO_2_/HCO_3_^−^, which caused a decrease in pH_i_ in both genotypes. *H*, BCECF loaded CPECs were acidified by superfusion with a 20 mm NH_4_Cl HEPES‐buffered solution (NH_4_Cl) followed by a washout in a CO_2_/HCO_3_^−^‐buffered Na^+^‐free solution (0Na^+^), dpHi/d*t* was determined following addition of 145 mm Na^+^ (Na^+^) in the presence of CO_2_/HCO_3_^−^ in NBCe2 KO (grey line) and WT (black line) mice. The experiment was ended by a 1‐point calibration in a high‐K^+^‐nigericin solution with pH 7.0 (not shown). *I*, mean values of the net acid efflux ± SEM estimated at the point of peak acidification (*n* = 5). ^*^Statistical significance (*P* < 0.05).

NBCe2 is known to export Na^+^ and HCO_3_^−^ from the CPE to the CSF with a 1:3 stoichiometry (Millar & Brown, 2008). To investigate the contribution of NBCe2 to base extrusion, the intracellular pH (pH_i_) recovery was monitored in SNARF‐loaded isolated choroid plexus cell clusters (Fig. 6 *A*). Intracellular pH (pH_i_) was calibrated in a high‐[K^+^] solution containing nigericin with a known extracellular pH (Table 3, Fig. 6 *B*). We have previously determined the intracellular buffering capacity for the choroid plexus in the low pH_i_ ranges with BCECF (pH 6.25–7.25) (Damkier *et al*. 2009). The total buffering capacity in the neutral‐to‐alkaline range is shown in Fig. 6 *C*. The contribution of the intrinsic buffering capacity at the alkaline pH range was negligible compared to the calculated buffering capacity arising from the CO_2_/HCO_3_^−^ buffer system (dashed line, Fig. 6 *C*). Intracellular pH was first determined in a HEPES‐buffered solution and switched to a CO_2_/HCO_3_^−^‐buffered solution (Fig. 6 *D*). This resulted in an initial transient drop in pH_i_ as a result of CO_2_ import followed by a more sustained increase in pH_i_ due to the import of HCO_3_^−^ into the cell. The pH_i_ increase did not differ between the two genotypes (Fig. 6 *E*; WT *n* = 6, KO *n* = 5, *P* = 0.62), indicating that the lack of NBCe2 does not result in altered baseline HCO_3_^−^ transport. The cells were then alkalinized by removal of Cl^−^ from the CO_2_/HCO_3_^−^‐buffered solution (Table 3). Peak pH_i_ after alkalization was similar in the two genotypes (NBCe2 WT 7.87 ± 0.04, KO 7.81 ± 0.06, *n* = 6, *P* = 0.36). The pH_i_ recovery rate after alkalization was determined as the pH_i_ change in the first 20 s after peak alkalization, where the NBCe2 activity is expected to be highest. The mean net base efflux was indeed significantly higher in the NBCe2 WT mice compared to NBCe2 KO mice (Fig. 6 *F*; WT *n* = 6, KO *n* = 5, *P* = 0.02). The net base efflux was also investigated in BCECF‐loaded CP cells alkalized with 20 mm tetramethylamonia (TMA). CP cells from WT mice had similar total base efflux, as well as numerically higher DIDS‐sensitive base efflux compared to CP cells from NBCe2 KO mice, but the difference was statistically insignificant (not shown).

Na^+^‐dependent HCO_3_^−^ extrusion at baseline pH_i_ was determined in BCECF‐loaded clusters of isolated CP by removing Na^+^ from the CO_2_/ HCO_3_^−^‐buffered solution. Baseline pH_i_ in the absence of CO_2_/HCO_3_^−^ did not differ between the genotypes (Fig. 6 *G* (HBS); WT *n* = 5, KO *n* = 6, *P* = 0.88). Like the experiments using SNARF, the intracellular response to switching from the HEPES‐buffered solution to a CO_2_/HCO_3_^−^‐buffered solution was similar between the genotypes (Fig. 6 *G*; CO_2_‐induced decrease (CO_2_ init.): *P* = 0.19; BBS: *P* = 0.67). The acidification rate induced by the removal of Na^+^ did not differ between genotypes (Fig. 6 *G*; *P* = 0.48).

Another way to detect the significance of outward transport of NBCe2 function is to assess the effect of NBCe2 deletion on net acid extrusion. In case NBCe2 activity normally opposes the transport activities of the acid extruders in the CPE, the net acid efflux from acidified cells should be augmented in the isolated choroid plexus cells from NBCe2 KO mice. Cells were loaded with BCECF and acidified. The effect of NBCe2 deletion on net acid extrusion was assessed by loading CPECs with BCECF and acidifying using an NH_4_Cl prepulse followed by a washout in Na^+^‐free CO_2_/HCO_3_^−^‐buffered solution (Fig. 6 *H*). Introducing Na^+^ in the continued presence of CO_2_/HCO_3_^−^ allowed the determination of the Na^+^‐dependent pH_i_ recovery rate. This value was determined as the change in pH_i_ during the first 20 s after addition of Na^+^. The mean Na^+^‐dependent acid efflux was 6‐fold larger in the CPE from NBCe2 KO mice compared to WT mice (Fig. 6 *I*). This indicates that the contribution of the base importers such as the HCO_3_^−^ importers Ncbe and NBCn1 is increased in acidified cells when NBCe2 is absent. Taken together, the results indicate that NBCe2 is an important base extruder at high pH_i_, whereas at baseline and low pH_i_, the contribution of NBCe2 to base extrusion is insignificant. Augmented pH_i_ recovery after intracellular acidification suggests increased activity of other HCO_3_^−^ importers.

### NBCe2 KO and WT mice display similar respiratory response to 5% CO_2_

**Figure 7:**
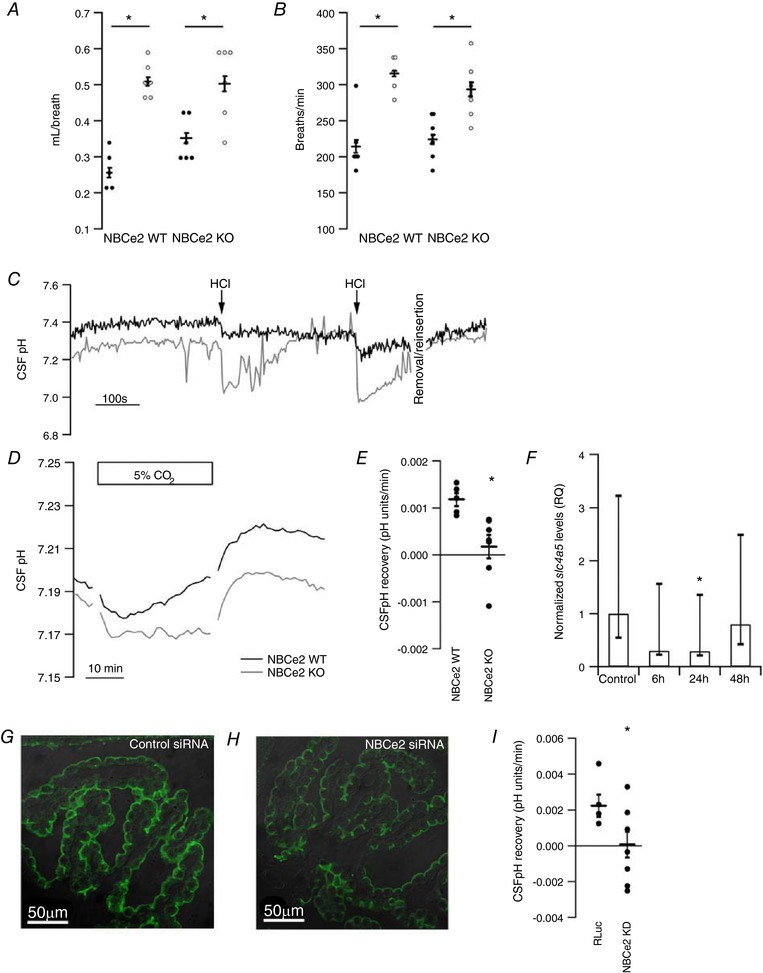
NBCe2 KO mice are deficient in CSF pH restoration during hypercapnia Tidal volume (*A*) and respiration frequency (*B*) were determined in atmospheric air (filled circles) and during inhalation of 5% CO_2_ (open circles) in NBCe2 KO and WT mice to ascertain that the two genotypes had similar ventilatory responses to 5% CO_2_ exposure (*n* = 6). *C*, validation of intraventricular CSF pH recording (raw data). Glass electrodes (grey line) or optical electrodes (black line) were inserted into the lateral ventricles of anaesthetized mice. Arrows indicate the times of injection of 1 μl 5 mm HCl into the contralateral ventricle. *D*, representative traces of *in vivo* CSF pH recordings before, during and after inhalation of 5% CO_2_ in NBCe2 KO (grey line) and WT (black line) mice. Graphs show pH values averaged at pH/min. *E*, mean values ± SEM CSF pH recovery rate during the last 20 min of the recovery phase during inhalation of 5% CO_2_ in NBCe2 KO (*n* = 6) and WT (*n* = 4). *F*–*H*, verification of siRNA knockdown. The efficiency of the siRNA approach to knock down NBCe2 was assessed by qPCR and semi‐quantitative immunohistochemistry. *F*, bar graph depicting the *Slc4a5* mRNA expression after 6 h, 24 h and 48 h after *Slc4a5* targeted siRNA relative to control mice injected with *RLuc*, as indicated. Bars represent relative quantification calculated by the comparative Ct method ± RQ min and max (*n* = 4). *G*, representative example of immunohistochemical staining for NBCe2 in control (*RLuc*) siRNA‐injected mice 48 h after treatment. *H*, similar image exemplifying staining for NBCe2 obtained 48 h after NBCe2 siRNA injection. *I*, graph showing mean values ± SEM of CSF pH recovery rate during the last 20 min of the recovery phase of 5% CO_2_ inhalation in mice injected with siRNA targeting *Slc4a5* mRNA (NBCe2 KD, *n* = 7) or the control *RLuc* (*n* = 5). ^*^ *P* < 0.05.

Respiratory frequency and tidal volume were determined during hypercapnia in NBCe2 KO and WT mice using barometric measurements. Inhalation of 5% CO_2_ elicited significant increases in tidal volume (Fig. 7 *A*) and respiratory frequency (Fig. 7 *B*) in both the NBCe2 WT and KO mice. There was no statistically significant difference between the genotypes in tidal volume or respiratory frequency either under baseline conditions or during 5% CO_2_ exposure (*n* = 6, *P* = 0.17 and *P* = 0.09, respectively). Under baseline conditions NBCe2 KO mice displayed normal plasma pH compared to WT (NBCe2 WT: 7.43 ± 0.02 *versus* NBCe2 KO: 7.43 ± 0.01 pH units, *P* = 0.88). The P aC O2 and standard HCO_3_^−^ (stHCO_3_^−^) were, however, reduced in knockout compared to wild type (P aC O2: NBCe2 WT 4.4 ± 0.3 kPa, NBCe2 KO 3.4 ± 0.3 kPa, *P* = 0.035; stHCO_3_^−^: NBCe2 WT 23.1 ± 0.5 mm, 20.2 ± 0.7 mm, *n* = 5, *P* = 0.004; WT *n* = 15, KO *n* = 10).

### NBCe2 KO and NBCe2 KD attenuate CSF pH recovery during acute respiratory acidosis

To investigate the role of NBCe2 in regulating CSF pH during acidification, pH sensors were placed in the lateral ventricles of anaesthetized mice. Figure 7 *C* shows examples of CSF pH traces obtained in NBCe2 WT mice before and after intraventricular injection of HCl and during a manoeuvre for retracting and reintroducing the pH sensor in the ventricles. A transient and reproducible decrease in CSF pH was observed upon HCl injection with both glass pH electrodes and the optical microsensor, while the removal and reintroduction of the electrode or sensor did not affect the recordings. Inhalation of 5% CO_2_ causes a respiratory acidosis, which is known to directly affect CSF pH (Wichser & Kazemi, 1975; Nattie & Edwards, 1981). Thus, NBCe2 WT and KO mice were subjected to 30 min inhalation of 5% CO_2_ while CSF pH was recorded (Fig. 7 *D*). There was no significant difference in baseline CSF pH between the NBCe2 WT and KO mice (NBCe2 WT 7.19 ± 0.24 (*n* = 5), NBCe2 KO 7.18 ± 0.20 (*n* = 7), *P* = 0.95). The deflections in CSF pH upon introduction and removal of 5% CO_2_ were also similar between the genotypes (pH decrease: NBCe2 WT −0.018 ± 0.006 pH units, NBCe2 KO −0.013 ± 0.002 pH units, *P* = 0.34; pH increase: NBCe2 WT 0.023 ± 0.003 pH units, NBCe2 KO 0.027 ± 0.003 pH units, *P* = 0.39). After the rapid CO_2_‐induced CSF pH decrease, a slow pH recovery was observed only in the NBCe2 WT mice (Fig. 7 *D*). The CSF pH recovery rate was determined over 20 min after maximal acidification and was significantly higher in NBCe2 WT mice than in NBCe2 KO mice (Fig. 7 *E*; *n* = 5 for WT, *n* = 7 for KO, *P* = 0.01).

To support the observations from the NBCe2 KO model and to rule out effects of NBCe2 disruption elsewhere in the body, NBCe2 was targeted specifically in the choroid plexus and circumventricular tissues by intraventricular installation of *Slc4a5* siRNA 48 h prior to the CSF pH measurements. Figure 7 *F* shows that mice injected with siRNA targeting NBCe2 had reduced abundance of NBCe2 mRNA after 24 h compared to controls (*RLuc* siRNA) by qPCR analysis (*n* = 4, *P* = 0.0497), whereas the NBCe2 mRNA level in NBCe2 siRNA‐injected mice after 6 and 48 h was not different from those observed in controls (*n* = 4, n.s.). At the protein level, however, a reduction of luminal membrane NBCe2 abundance was observed 48 h after siRNA injection. Immunohistochemical staining of mouse CPE from a similar experiment 48 h after siRNA injection targeting *RLuc* and *Slc4a5* (NBCe2) is seen in Fig. 7 *G* and *H*, respectively. Semiquantitative analysis of the fluorescence intensity corresponding to NBCe2 protein levels in the siRNA‐injected mice indicated approximately 60% knockdown of the protein after 48 h (*n* = 2). Based on the protein analysis, 48 h post‐injection was chosen as the time for the functional measurements. Similar to the NBCe2 KO mice, NBCe2 KD mice did not have significant differences in baseline CSF pH (control pH = 7.36 ± 0.07, *n* = 9; 24 h after injection, pH = 7.35 ± 0.09, *n* = 8; 48 h after injection pH = 7.34 ± 0.05, *n* = 6). The NBCe2 KD mice presented with a practically abolished CSF pH recovery during acidosis compared to control mice injected with *RLuc* siRNA (Fig. 7 *I*; *P* = 0.044, *n* = 5 for WT, *n* = 8 for KD).

### NBCe2 KO mice are not protected against seizure development

**Figure 8:**
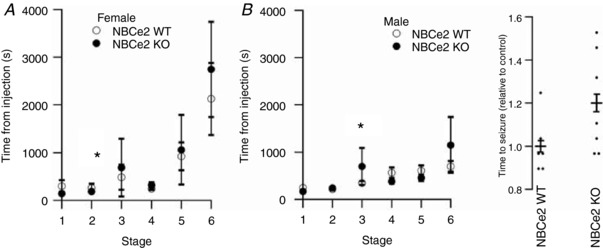
NBCe2 KO mice are not protected against experimental seizures Mice were injected intraperitoneally with pentylenetetrazol (PTZ) and observed for up to 60 min. Seizure activity was determined as the time from injection of PTZ to development of first appearance in each seizure activity score (see text for details) in female (*A*) and male (*B*) NBCe2 KO and WT (*n* = 5). *C*, mice were subjected to hyperventilation‐induced seizure challenge induced by heating. The graph shows the mean lag time ± SEM between when heating was initiated and the first observed seizures (*n* = 7 for NBCe2 KO; *n* = 6 for WT).

During seizure attacks, brain interstitial pH is lowered due to local hypoxia. This lowering of pH promotes the disruption of the seizure, if the seizure is caused by alkaline brain pH, as for instance during febrile seizures (Schuchmann *et al*. 2006). Thus seizure activity seems dependent on the ability of the brain interstitial fluid to respond to acid‐base changes. As CSF pH in the NBCe2 KO mice shows a much smaller recovery in response to acidosis and isolated CP cells show inadequate base extrusion in response to alkalosis, NBCe2 KO mice could hypothetically be better protected against development of seizures. PTZ‐injected or heat‐treated mice were therefore videotaped and scored for seizure development as described in the Methods section. In general, there was no protection against PTZ‐induced seizures in NBCe2 KO mice as judged by the time course of the seizure development in females (*n* = 5; Fig. 8 *A*) or in males (*n* = 5; Fig. 8 *B*). However, the time to development of stage 3 seizures (myoclonic jerks/back arches) was significantly increased in male NBCe2 KO compared to NBCe2 WT mice (*P* = 0.0008). In the female group, NBCe2 KO mice displayed a statistically significant shorter time lag before entering stage 2 than NBCe2 WT mice, although the numerical values were very close (*P* = 0.04). In the same experiments, the maximal score obtained after 20 min was also equal between genotypes for males and females, but there seemed to be a tendency towards a gender difference in seizure score, with male mice reaching a higher score than females (mean scores: female NBCe2 WT 2.7 ± 0.85, female NBCe2 KO 3 ± 0,94; male NBCe2 WT 4.6 ± 0.24, male NBCe2 KO 4.5 ± 0.22, *P* = 0.12).

The time lag before seizure development in the heat‐treated hyperventilating mice tended to be longer for NBCe2 KO mice (Fig. 8 *C*; *n* = 6 for WT, *n* = 7 for KO, *P* = 0.0935). The physical activity in the escape behaviour, however, was significantly decreased in NBCe2 KO mice as assessed by the number of jumps per time unit (*P* = 0.0039).

## Discussion

By exploiting continuous *in vivo* CSF pH recording in a novel NBCe2 KO mouse model, and by specifically targeting NBCe2 in the brain using siRNA knockdown, we identify the first acid‐base transport protein involved in CSF pH regulation at the blood‐CSF barrier. Since Husted and Reed in 1977 showed that the CSF HCO_3_^−^ concentration is actively regulated by the CPE (Husted & Reed, 1977), it has been suggested that acid‐base transport processes in the CPE would be involved in regulating CSF pH. We hypothesized that NBCe2, being an efficient bicarbonate extruder in the CPE, might be a suitable mechanism for alkalizing CSF pH. We showed that NBCe2 is critically required to recover CSF pH during early respiratory acidosis, and that this effect is not caused by differences in gross phenotype, respiratory rate or tidal volume.

NBCe2 is known to export Na^+^ and HCO_3_^−^ with a 1:3 stoichiometry from the CPECs across the luminal (CSF‐facing) plasma membrane (Millar & Brown, 2008) and is therefore expected to be most active during e.g. extracellular acidification or intracellular alkalization at typical membrane potential values. Our results from intracellular pH measurements using SNARF at alkaline pH_i_ in NBCe2 KO mice demonstrate a decrease in net base excretion in the NBCe2 KO compared to wild‐type mouse CPE in the presence of CO_2_/HCO_3_^−^. The manoeuvre to alkalize the cells (Cl^−^ removal) has the advantage of preventing base extrusion by the anion exchanger AE2 during the pH recovery. In experiments with TMA alkalized BCECF‐loaded cells, we observed a numerical reduction in base extrusion in NBCe2 KO CPE that was sensitive to the stilbene derivative DIDS (4,4′‐diisothiocyano‐2,2′‐stilbenedisulfonic acid) although the difference was not statistically significant. The sensitivity of BCECF in the high pH_i_ range is lower than that of SNARF, which resulted in larger standard errors. Nevertheless, the numerical value of the total base efflux in NBCe2 WT CPE, as well as the residual base efflux (non‐NBCe2, non‐AE2), was highly similar between the two experimental approaches. Although the lack of significantly different values by the BCECF approach presents a weakness in our study, we believe that the results from the SNARF experiments, isolation of NBCe2 function by Cl^−^ removal and our *in vivo* data strongly indicate that NBCe2 is a Na^+^‐HCO_3_^−^ extruder thereby confirming previous studies (Millar & Brown, 2008). The contribution of NBCe2 to acid‐base regulation was investigated at baseline and in acidic conditions. In baseline conditions, addition of CO_2_/HCO_3_^−^ resulted in similar alkalization rate between genotypes. Furthermore, the removal of Na^+^ in the presence of CO_2_/HCO_3_^−^ resulted in a similar outward Na^+^‐driven HCO_3_^−^ transport in the two genotypes. In acidic conditions, the result was less easy to interpret. A base extruder is not likely to be active in acidic conditions, where the activity of the acid extruders is high (Parker & Boron, 2013). Removal of a base extruder as in the NBCe2 KO model would therefore not be expected to result in a difference in acid extrusion. Nevertheless, when comparing the pH_i_ recovery of isolated CPECs from NBCe2 WT and KO mice after acidification, the lack of the base extruder NBCe2 seems to greatly increase the apparent activity of the Na^+^‐dependent acid extruders, such as Ncbe, NBCn1 and NHEs. In the absence of compensatory changes in acid‐base transporter expression, we interpret the increased pH_i_ recovery rate as a *functionally* enhanced Na^+^‐dependent acid extrusion in NBCe2 KO CPE. Taken together, we verify NBCe2 as a base extruder in CPECs, which is detectable by pH_i_ recordings after alkalization. Although NBCe2 activity *per se* was observed at baseline pH_i_ by electrophysiological means (Millar & Brown, 2008), our baseline pH_i_ experiments suggest that NBCe2 is not involved in setting the resting pH_i_.

As mentioned above, a mechanism for import of base equivalents into the CSF was proposed to explain that the changes in CSF pH do not surpass the changes in plasma pH during systemic acid‐base disturbances, despite the lack of protein buffering in the CSF (Yuan & Desiderio, 2005). However, the molecular identity of the proteins mediating this transport across the blood‐brain barrier or the blood‐CSF barrier has remained elusive until now. To test whether NBCe2 is involved in CSF pH regulation it was necessary to establish a method for continuous CSF pH recording *in vivo*. To the best of our knowledge, this is the first time this method has been applied to assess CSF pH changes, although *in vivo* pH measurements of brain tissue (Schuchmann *et al*. 2006) and baseline CSF pH (Mani *et al*. 2017) have previously been performed. With this method, we were able to reliably detect the abrupt CSF pH changes upon HCl injections or hypercapnia, and measure the fast CSF pH compensations to these disturbances within minutes after inflicting the perturbations. We show that the recovery of CSF pH during hypercapnia‐induced acidification is greatly decreased in the NBCe2 KO mice, pointing to CPE NBCe2 as the major molecular mechanism underlying base extrusion during CSF acidification. Applying DIDS to the ventricle system would inhibit both NBCe2 and AE2, and seems an unattractive approach to verify the involvement of NBCe2 in CSF pH regulation (Deng & Johanson, 1989). In the absence of specific NBCe2 inhibitors, we developed siRNA‐mediated knockdown of the protein in order (1) to verify the involvement of NBCe2 in CSF pH regulation and (2) to exclude the influence of NBCe2 expressed outside the central nervous system. Similar to the NBCe2 KO mice, the NBCe2 KD mice failed to recover the CSF pH significantly during hypercapnia‐induced acidosis, confirming that reduced NBCe2 expression in the CPE causes a defect in CSF pH regulation from acidification. A similar observation was made for the electrogenic Na^+^‐HCO_3_^−^ cotransporter, NBCe1 in astrocytes (Theparambil *et al*. 2016). Astrocytes are suggested to secrete bicarbonate through NBCe1 in response to respiratory acidosis similar to what we observe for NBCe2 in this study. The altered CSF pH response was not caused by a difference in the respiratory response to inhalation of 5% CO_2_ but is a direct effect of the lack of local import of HCO_3_^−^. It is, however, unexpected that the ventilatory response to 5% CO_2_ is similar in the two genotypes given the lack of appropriate HCO_3_^−^ import into the CSF. This is most likely due to the acute nature of the experimental set‐up. The mice were only exposed to 5% CO_2_ for 5 min. During the first 5 min of CO_2_ exposure, we did not detect any difference in CSF pH in the two genotypes, which means that the central chemoreceptors are exposed to similar pH. Our baseline findings, however, suggest that the long‐term effect of lacking a HCO_3_^−^ transporter in the CPE leads to a longer lasting effect on the chemoreceptors that slightly increases the respiratory drive, leading to an increased washout of CO_2_. This will cause a respiratory alkalosis compensated by increased renal excretion of HCO_3_^−^. The acid‐base status of the NBCe2 KO mice is indeed indicative of a compensated acid‐base disturbance characterized by normal blood pH and lower P aC O2 and stHCO_3_^−^ compared to WT, similar to the study by Gröger *et al*. (2012). Gröger *et al*. show that the phenotype is caused by renal loss of HCO_3_^−^ causing an acidosis followed by a respiratory compensation. Our plethysmography experiments, however, show a similar respiration rate in the knockout compared to wild type under baseline conditions. This is surprising if the mice indeed have a metabolic acidosis with respiratory compensation. Further studies are needed to isolate the renal *versus* the central effect on the acid‐base disturbance.

Dysregulation of brain pH has been linked to altered seizure susceptibility (Schuchmann *et al*. 2006; Ziemann *et al*. 2008). In a study by Kao and coworkers, a gene‐trap deletion of NBCe2 resulted in increased seizure threshold in mice following injections with the proconvulsant drug PTZ (Kao *et al*. 2011). By contrast, PTZ injections in our NBCe2 KO mice show only minor if any differences among genotypes. The onset of seizure development in NBCe2 KO mice was lower at only one of the six stages, indicating that the NBCe2‐deficient mice are generally not protected against development of seizures. Another way to induce seizures is to increase brain pH by hyperventilation, as, for example, induced by exposing the mice to elevated ambient temperature. Although there is a numerical tendency towards protection in NBCe2 KO mice, we do not detect a statistically significant difference in the time lag before seizure development in these experiments. The observation that NBCe2 KO mice were less active during the experiment complicates the interpretation of the results, as the increased activity would lead to increase in respiratory rate and thereby further brain alkalization in the NBCe2 wild‐type compared to KO mice. Further studies are, therefore, necessary to explore the difference in activity we observe in the hyperventilation experiments.

The gene‐trap NBCe2 KO mouse described by Kao *et al*. displayed alterations in the localization of several membrane transporters and cytoskeletal proteins in the CPE (Kao *et al*. 2011), as described in the introduction. In contrast, our knockout model exhibits an unchanged expression pattern of solute transporters, such as the Na^+^,K^+^‐ATPase (α1 or β1 subunit), AQP1, and NKCC1. Although our pH_i_ measurements indicate increased functional activity of acid extruders at low pH_i_, the expression and localization of the HCO_3_^−^ transporters Ncbe, NBCn1 and AE2 are similar in the two genotypes, as was the localization pattern of the spectrin cytoskeleton in our study. The potential seizure protective effect of NBCe2 KO presents a major discrepancy between the current study and the gene‐trap study by Kao and coworkers. Whereas the gene‐trap NBCe2 KO mouse was protected against seizures (Kao *et al*. 2011), we found little evidence for such protection by the same method and scoring system in our model, although the underlying hypothesis was very appealing. The brain ventricle volume in the gene trap model was decreased, while the exon 7 deletion approach by Gröger *et al*. did not result in brain ventricle volume changes (Gröger *et al*. 2012). In addition to the frame shift also applied in the two previous models, our approach targets the conserved first transmembrane segments of NBCe2 to prevent signal peptide mediated transfer into RER and eventually plasma membrane insertion. Thus, our model gives rise to a truncated N‐terminal part of the protein, which is undetectable even with an antibody directed against an N‐terminal epitope. Therefore, we are confident that our NBCe2 deletion has minimal cellular effects compared to the gene‐trap model. It would be very interesting to perform direct comparative physiological studies with these three NBCe2 models. Although we did not determine brain ventricle volume in our knockout mouse, we would expect a similar result as the mouse described by Gröger *et al*. since the expression of the transporters involved in CSF secretion are unaffected.

In conclusion, our study provides the first evidence of a specific transport protein harboured in the luminal membrane of the CPE to be directly involved in CSF pH regulation. We show that the sodium bicarbonate cotransporter NBCe2 is critically involved in CSF pH recovery during hypercapnia‐induced acidosis, which might protect the brain from acid‐induced injury.

## Additional information
